# Testosterone Contributes to Vascular Dysfunction in Young Mice Fed a High Fat Diet by Promoting Nuclear Factor E2–Related Factor 2 Downregulation and Oxidative Stress

**DOI:** 10.3389/fphys.2022.837603

**Published:** 2022-03-08

**Authors:** Rafael M. Costa, Rhéure Alves-Lopes, Juliano V. Alves, Carolina P. Servian, Fabíola L. Mestriner, Fernando S. Carneiro, Núbia de S. Lobato, Rita C. Tostes

**Affiliations:** ^1^Department of Pharmacology, Ribeirão Preto Medical School, University of São Paulo, Ribeirão Preto, Brazil; ^2^Academic Unit of Health Sciences, Federal University of Jatai, Jatai, Brazil; ^3^British Heart Foundation, Glasgow Cardiovascular Research Centre, Institute of Cardiovascular and Medical Sciences, University of Glasgow, Glasgow, United Kingdom

**Keywords:** obesity, testosterone, vascular dysfunction, Nrf2, oxidative stress

## Abstract

Obesity, an important risk factor for cardiovascular disease, promotes vascular oxidative stress. Considering that free testosterone levels remain within the reference range, especially in obese young men and that testosterone stimulates reactive oxygen species (ROS) generation, we sought to investigate whether testosterone interferes with obesity-associated oxidative stress and vascular dysfunction in male mice. We hypothesized that testosterone favors ROS accumulation and vascular dysfunction in high fat diet (HFD)-fed obese mice. We also questioned whether testosterone downregulates the nuclear factor E2–related factor 2 (Nrf2), one of the major cellular defense mechanisms against oxidative stimuli. Male C57Bl/6J mice were submitted to orchiectomy or sham-operation. Mice received either a control diet (CD) or HFD for 18 weeks. Vascular function was assessed in thoracic aortic rings and molecular mechanisms by which testosterone contributes to vascular dysfunction were determined. HFD reduced acetylcholine-induced vasodilation and increased vascular ROS generation in sham mice. Castration prevented these effects. Treatment of castrated mice fed either the CD or HFD with testosterone propionate decreased acetylcholine vasodilation. HFD decreased Nrf2 nuclear accumulation, events linked to decreased mRNA expression and activity of Nrf2-regulated enzymes (catalase, heme oxygenase-1, peroxiredoxin, and thioredoxin). These events were prevented in HFD-fed castrated mice. Bardoxolone, a Nrf2 activator, increased nuclear accumulation of Nrf2, decreased ROS generation and improved acetylcholine vasodilation in HFD-fed sham mice. *In vitro*, testosterone increased ROS generation and decreased Nrf2 nuclear accumulation. These effects were prevented in the presence of an androgen receptor antagonist, an inhibitor of gene transcription and an inhibitor of the pro-oxidant enzyme NOX-1. These results indicate that testosterone downregulates Nrf2, leading to oxidative stress and vascular dysfunction in HFD-fed obese young mice.

## Highlights

– Testosterone contributes to vascular dysfunction in young obese.
– Vascular dysfunction in young obese is associated with testosterone-induced oxidative stress.
– Testosterone contributes to decreased Nrf2 transcriptional factor signaling, compromising the antioxidant function in the vascular system.
– The decrease in testosterone levels during the development of obesity in young individuals attenuates oxidative stress, improving the performance of the transcription factor Nrf2 and consequent prevention of vascular dysfunction.

## Introduction

Obesity is a major cause of morbidity and mortality in a large part of the world population (Andersson and Vasan, 2018; Csige et al., 2018). Numerous comorbid conditions are associated with adult obesity, including type 2 diabetes mellitus, arterial hypertension, and other cardiovascular and metabolic disorders (Krauss et al., 1998; Kahn et al., 2006; Jiang et al., 2016; Costa et al., 2018). Excessive fat accumulation is widely considered the result of interactions between environmental, genetic and epigenetic factors, which disturb the balance between the caloric intake and energy expenditure, leading to increased risk of illness (Hill, 2006). The prenatal period and the first years of an individual’s life were identified as critical phases for the development of body composition (Stettler and Iotova, 2010). High birth weight is associated with an increased risk of obesity in adulthood (Yu et al., 2011; Zhao et al., 2012). Furthermore, faster weight gain in children is also associated with an increased risk of overweight and obesity later in life (Monteiro and Victora, 2005; Ong and Loos, 2006; Druet et al., 2012). Fat deposition in childhood and early adulthood also accounts for increased cardiovascular risk (Nadeau et al., 2011; Caprio et al., 2020). However, it is unclear how body mass gain during the transition from early to middle adulthood, when most body mass gain occurs, relates to subsequent health consequences.

One important factor that has been associated with both childhood and adult obesity are testosterone levels (Kelly and Jones, 2013; Mancini et al., 2021). Nevertheless, little attention has been directed toward the role of androgenic steroids on the mechanisms of cardiovascular and metabolic alterations in obesity. Although still a controversial topic, androgens are widely associated with higher risk of cardiovascular disease in men. In fact, abusive use of synthetic androgens by athletes leads to premature cardiovascular complications and the male gender itself is an independent risk factor for cardiovascular disease (Kloner et al., 2016). In addition, there is abundant epidemiological and clinical evidence linking a decline in testosterone levels with vascular dysfunction, atherosclerotic process, stroke and cardiovascular mortality (Bhasin and Herbst, 2003; Lopes et al., 2012; Alves et al., 2020). One explanation for the lack of agreement amongst these studies is that testosterone may shift target tissues susceptibility to damage by mechanisms that depend on the concentration, duration and source (exogenous androgen, salt of testosterone), as well as the age, sex and health/disease state of the individual undergoing testosterone treatment.

Oxidative stress, a disturbance in the balance between the production of free radicals and antioxidant defenses, plays a critical role in the pathophysiology of obesity and obesity-associated complications (Betteridge, 2000). Obesity itself induces systemic oxidative stress through mechanisms that involve superoxide anion generation from NADPH oxidases, oxidative phosphorylation, and chronic inflammation. Adipose tissue dysfunction in obesity leads to increased reactive oxygen species (ROS) generation and inadequate cellular antioxidant defense (Furukawa et al., 2004; Zhou et al., 2021). In the cardiovascular system, oxidative stress occurs concomitantly with endothelial dysfunction, increased vascular contractility, growth and apoptosis of vascular smooth muscle cells (VSMC), lipid peroxidation and increased extracellular matrix protein deposition (da Costa et al., 2017; Costa et al., 2018). Testosterone and its metabolites affect the main components that control/modulate ROS generation and degradation (Tostes et al., 2016). Testosterone induces ROS generation in VSMC via NADPH oxidases, with greater production in cells from hypertensive compared with normotensive animals (Chignalia et al., 2012). Testosterone also induces mitochondria-derived ROS generation and apoptosis in VSMC, effects mediated via androgen receptor activation (Lopes et al., 2014). Testosterone stimulates the generation of superoxide anion (via xanthine oxidase) and nitric oxide, increasing peroxynitrite production and stimulating apoptosis (Puttabyatappa et al., 2013). Cyclooxygenase2-dependent ROS production is obligatory for testosterone-induced immune cells migration (Chignalia et al., 2015), which may contribute to inflammation and target organ damage. Collectively, these data indicate that in oxidative conditions, androgen signaling further increases cell damage.

Antioxidants are compounds or enzymatic systems that scavenge or neutralize free radicals (Kawagishi and Finkel, 2014). In this sense, the nuclear factor E2–related factor 2 (Nrf2) is one of the major defense mechanisms against oxidative and proteotoxic stress in cells (Cuadrado et al., 2019). Under physiological conditions, Nrf2 is usually associated with Keap-1, a repressor protein found in the cytoplasm (Horie et al., 2021). Stressors, such as free radicals, favor the translocation of Nrf2 to the cell nucleus. The accumulation of nuclear Nrf2 allows its binding to the antioxidant response element of genes that code antioxidant proteins (Nguyen et al., 2009). Growing evidence indicates that decreased or defective Nrf2 activity contributes to oxidative stress, favoring the pathophysiology of cardiovascular disorders found in obesity (da Costa et al., 2019).

While it is clear that testosterone regulates ROS generation, it is undefined whether male sex hormones contribute to the progressive vascular oxidative damage caused by obesogenic diets. In this study, we questioned whether loss of androgens in young male mice changes the tissue’s hormonal landscape, yielding a vascular oxidative status that differs from that observed in adult males. Accordingly, we used castrated young male mice to determine whether androgens confer protection or exacerbate vascular oxidative damage induced by an obesogenic diet. We hypothesized that testosterone, signaling through androgen receptors, negatively impacts Nrf2/Keap1 signaling. We also determined whether testosterone treatment changes Nrf2/Keap1 signaling. Finally, we determined *in vitro* effects of testosterone on ROS generation and Nrf2 nuclear accumulation, using endothelial cells treated with vehicle or the androgen receptor antagonist flutamide. Alternatively, the functional and molecular components were measured in the presence of vehicle or the Nrf2 activator bardoxolone.

## Materials and Methods

### Animals, Orchiectomy, and Diets

All experimental protocols were performed in accordance with the recommendations of the Brazilian Guidelines for the Care and Use of animals for Scientific and Teaching Purposes and the Guidelines for the Practice of Euthanasia (CONCEA-MCT, 2013), having been approved by the Ethics Committee on Animal Use of the University of São Paulo, Ribeirão Preto, Brazil (Protocol n° 206/2016).

Four-week-old male C57Bl/6J mice were obtained and maintained in the Animal Facility of the University of São Paulo, Ribeirão Preto, Brazil on 12-h light/dark cycles under controlled temperature (22 ± 1°C) and humidity (50–60%) with *ad libitum* access to food and water. Mice were anesthetized (ketamine and xylazine at doses of 75 and 15 mg/kg, respectively, intraperitoneally), the efferent duct of each testicle was ligated, and the testicles were removed. Sham mice were submitted to the same surgical procedures without efferent duct ligation or testicles removal. After a 1-week acclimatization period, mice were divided into two groups: (1) mice maintained on a control diet [CD (protein 22%, carbohydrate 70%, and fat 8% of energy, PragSolucoes)], and (2) mice receiving a high-fat diet [HFD (protein 10%, carbohydrate 25%, and fat 65% of energy, PragSolucoes)]. After 18 weeks on the CD or HFD, mice were euthanized by carbon dioxide (CO_2_) inhalation without removing mice from their home cage. Death was confirmed by cardiac arrest. Studies followed the ARRIVE guidelines for reporting experiments on experimental animals.

### Biochemical Profile

Glucose levels were determined using a glucose analyzer (Accu-Check, Roche Diagnostics). Total cholesterol and triglyceride concentrations were determined in serum samples from mice fasted for 12 h (da Costa et al., 2018), by enzymatic colorimetric method (Doles^®^). Plasma insulin concentration (ng/mL) was determined by radioimmunoassay (Insulin Kit^®^).

### Testosterone Replacement and Plasma Measurement

Plasma testosterone levels were determined in sham, castrated and testosterone-treated mice using the IMMULITE 1000 Immunoassay System (Enzo Life Sciences^®^, New York, NY, United States). After 18 weeks on the CD or HFD, testosterone-treated mice received intramuscular injections of testosterone-propionate [dissolved in peanut oil, 5 mg/kg body weight per day (Sigma Aldrich, San Luis, MO, United States)], for 15 days.

### Assessment of Vascular Function

Thoracic aorta was rapidly removed, transferred to an ice-cold (4°C) Krebs Henseleit-modified solution [(in M) 130 NaCl, 14.9 NaHCO_3_, 4.7 KCl, 1.18 KH_2_PO_4_, 1.17 MgSO_4_⋅7H_2_O, 5.5 glucose, 1.56 CaCl_2_⋅2H_2_O and 0.026 EDTA] gassed with 5% CO_2_ and 95% O_2_ to maintain a pH of 7.4, and dissected into 2 mm rings. Aortic rings were mounted in a wire myograph to measure isometric tension, as previously described (da Costa et al., 2017). Vessels were allowed to equilibrate for about 30 min in Krebs Henseleit solution. After the stabilization period, endothelial function was assessed by testing the relaxant effect of acetylcholine (ACh, 10^–6^ M) on vessels contracted with phenylephrine (PE, 10^–7^ M). Aortic rings exhibiting a vasodilator response to ACh greater than 70–80% were considered endothelium-intact vessels. Cumulative concentration-response curves to ACh (10^–10^ to 10^–4^ M) were performed in aortic rings from the various experimental groups.

Relaxation responses to ACh were also determined after incubation with either the selective superoxide anion (O_2_^–^) scavenger tiron (10^–4^ M, 30 min) or the Nrf2 activator bardoxolone (10^–6^ M, 3 h). Each vascular preparation was tested with a single agent.

### Cultured Endothelial Cells and Experimental Design

Endothelial cells were commercially purchased. An immortalized line of human umbilical vein endothelial cells, EA.hy926, (ATCC^®^ CRL-2922™) was used. Cells were cultured in Dulbecco’s Modified Eagle Medium (DMEM) supplemented with 10% fetal bovine serum (FBS) in a CO_2_ incubator, at 37°C, 5% CO_2_. After confluence, cells were maintained in the presence of 2% FBS (Rodrigues et al., 2021). Cells were stimulated with testosterone for various times (10^–7^ M, 15 min to 6 h). To determined testosterone effects on NADPH oxidase activity, cells were preincubated with a NOX1 inhibitor ML171 (10^–4^ M, 1 h). To investigate genomic and non-genomic effects of testosterone, cells were incubated with the androgen receptor antagonist flutamide (10^–5^ M, 1 h) and the gene transcription inhibitor actinomycin D (10^–5^ M, 1 h), respectively, previous to exposure to testosterone.

### Measurement of Reactive Oxygen Species

#### Dihydroethidium

Reactive Oxygen Species generation was assessed by dihydroethidium (DHE), as previously described (Costa et al., 2016). Aortas were embedded in the optimal cutting temperature (OCT™) embedding medium and stored at −80°C. Fresh-frozen specimens were cross-sectioned at 10 μm thickness and placed on slides covered with poly-(L-lysine). Tissues were loaded with the non-selective dye for ROS detection DHE (5 × 10^–6^ M in phosphate buffer 10^–6^ M) for 30 min at 37°C. Images were collected on a ZEISS microscope and the results are expressed as fold changes relatively to the control. Fluorescent images were analyzed by measuring the mean optical density of the fluorescence in a computer system (Image J software) and normalized by the area.

#### Amplex Red

Thoracic aortae were frozen, macerated and centrifuged. Fifty μL-aliquots of the supernatant were removed and hydrogen peroxide (H_2_O_2_) was determined fluorometrically by measuring Amplex Red (8 × 10^–6^ M) conversion (Molecular Probes, Invitrogen, Carlsbad, CA, United States) to the fluorescent compound resorufin, in the presence of horseradish peroxidase (4 U/mL). Resorufin fluorescence was detected in a plate fluorimeter (Synergy™ 2 Multi-Detection Microplate Reader, BioTek Instruments, Santa Clara, CA, United States) using excitation and emission wavelengths of 530 and 590 nm, respectively. The fluorescence values were normalized by the total amount of tissue proteins.

#### Lucigenin

Generation of O_2_^–^ was measured by lucigenin luminescence using NADPH as the substrate. Aortae from control and obese mice were homogenized in an assay buffer (50 mM KH_2_PO_4_, 1 mM EGTA, and 150 mM sucrose, pH 7.4) with a glass-to-glass homogenizer. 100 μL of sample, lucigenin (5 μM), NADPH (0.1 mM) and assay buffer were used. Luminescence was measured for 30 cycles of 18 s each, in a luminometer (Orion II Luminometer, Berthold Detection Systems, Pforzheim, Germany). Basal readings were obtained and the reaction was started by the addition of the substrate. Basal and buffer blank values were subtracted from the NADPH-derived luminescence. O_2_^–^ was expressed as relative luminescence units (RLU)/mg protein.

### Measurement of Thiobarbituric Acid Reactive Substances

Thiobarbituric acid reactive substances (TBARS) concentration was determined colorimetrically (at 540 nm) in aortas homogenates with a commercially available kit (#10009055, Cayman Chemical, United States), following the manufacturer’s instructions. TBARS concentration was determined using a standard curve of malondialdehyde and the results are expressed as μM/mg protein.

### NO*x*

Plasma nitrate concentrations were determined by subtracting the nitrite values from the NO*x* values (nitrite + nitrate). For the determination of NO*x*, the Griess reaction was used. Plasma samples were incubated with nitrate reductase, at 37°C for 12 h, in a light-free environment. The Griess reagent was added and reading was performed by spectrophotometry at 540 nm.

### Thioredoxin Reductase, Superoxide Dismutase, and Catalase Activities

Thioredoxin reductase (TrxR) activity was determined in aortas using a commercially available kit (#CS0170, Sigma-Aldrich, San Luis, MO, United States). Enzymatic activity was assessed by determining the difference between the time-dependent increase in absorbance at 412 nm in the presence of the TrxR activity inhibitor from total activity. One activity unit equaled 1 mM 5′-thionitrobenzoic acid formed/mg protein. Thoracic aortae were homogenized in 300 μL of Krebs Henseleit and centrifuged at 18,000 × *g* for 15 min at 4°C. The supernatants were analyzed for superoxide dismutase (SOD) or catalase (CAT) activities. SOD activity was evaluated using a commercially available kit (#19160, Sigma-Aldrich, San Luis, MO, United States). The results were normalized for protein concentration, and SOD activity is expressed as % inhibition rate/mg protein. CAT activity was assayed by H_2_O_2_ consumption and measured in a spectrophotometer at 240 nm. One CAT unit (U) was defined as the amount of enzyme required to decompose 1 μM of H_2_O_2_/min/mg protein.

### Nuclear Factor E2–Related Factor 2 Activity

To determine nuclear accumulation of Nrf2, the nuclear fraction from cell or tissue lysates were separated using the Active Motif nuclear extract kit (Active Motif, Carlsbad, CA, United States) following the manufacturer’s protocol. Cells or tissue were resuspended in 1X hypotonic buffer and centrifuged for 30 s at 12,800 × *g* at 4°C. Nuclear pellets were resuspended in lysis buffer provided by the manufacturer. The suspension was incubated for 30 min on ice on a rocking platform set at 5 × *g* and then centrifuged for 10 min at 12,800 × *g*. The supernatant was transferred to a pre-chilled microcentrifuge tube. TransAM™ Nrf2 ELISA kit (Active Motif) was used to measure nuclear accumulation of Nrf2 at a wavelength of 450 nm.

### Quantitative Real-Time Reverse Transcription–Polymerase Chain Reaction

Total RNA was isolated from aortae using Trizol^®^ (Invitrogen, Carlsbad, CA, United States). RNA was treated with DNAse I (1 U/μL, Promega) and used for first-strand cDNA synthesis, accordingly to the manufacturer instructions. mRNA levels were quantified in triplicate by qPCR StepOnePlus™ *Life Technologies*. Specific primers (*TaqMan*™) for RT-qPCR were as follows: mouse SOD-1 *[Mm01344233_g1]*, Catalase *[Mm00437992_m1]*, Heme oxygenase-1 *[Mm00516005_m1]*, Peroxiredoxin-1 *[Mm01621996_s1]* and β-actin *[Mm00607939_s1]*, purchased from *Life Technologies*. PCR cycling conditions included 10 min at 95°C, followed by 40 cycles at 95°C for 15 s, 60°C for 1 min, and 72°C for 60 s. Dissociation curve analysis confirmed that signals corresponded to unique amplicons. Specific mRNA expression levels were normalized relatively to β-actin mRNA levels using the comparative ΔΔCt method.

### Western Blot Analysis

Aortas were frozen in liquid nitrogen and homogenized in a lysis buffer [50 mM Tris/HCl, 150 mM NaCl, 1% Non-idet P40, 1 mM ethylenediaminetetraacetic acid (EDTA), 1 μg/mL leupeptin, 1 μg/mL pepstatin, 1 μg/mL aprotinin, 1 mM sodium orthovanadate, 1 mM phenylmethylsulphonyl fluoride (PMSF), and 1 mM sodium fluoride]. The tissue extracts were centrifuged, and total protein content was quantified using the Bradford method (Bradford, 1976). Proteins (40 μg) were separated by electrophoresis on 10% polyacrylamide gel, and transferred on to nitrocellulose membranes. Non-specific binding sites were blocked with 5% bovine serum albumin (BSA) in Tris-buffered saline (TBS) containing 0.1% Tween 20 (for 1 h at 24°C). Membranes were incubated with antibodies (at the indicated dilutions) overnight at 4°C. Antibodies were used as follows: anti-keap1 (1:1,000 dilution; Abcam), anti-NOX1 (1:1,000 dilution; Cell Signaling), anti-NOX4 (1:1,000 dilution; Cell Signaling), anti-βactin (1:1,0,000 dilution; Sigma), and anti-GAPDH (1:10,000 dilution; Sigma). After incubation with secondary antibodies, signals were obtained by chemiluminescence, visualized by autoradiography and quantified densitometrically.

### Data and Statistical Analyses

The individual concentration–response curves were fitted into a curve by non-linear regression analysis. *p*D_2_ (defined as the negative logarithm of the EC_50_ values) and maximal response (Emax) were compared by Two-way ANOVA with Bonferroni post-test. The results of the molecular experiments were analyzed by Mann Whitney test or Two-way ANOVA, followed by the Bonferroni post-test. Data were assessed for normality with Shapiro-Wilk test. The Prism software, version 9.0 (GraphPad Software Inc., San Diego, CA, United States) was used to analyze these parameters as well as to fit the sigmoidal curves. Data are presented as mean ± SEM. *N* represents the number of mice used. *p* values less than 0.05 were considered significant.

## Results

### The High Fat Diet Effects on Adiposity in Young Mice Is Not Under the Influence of Testosterone

Table 1 details the characteristics of the animal groups. At the beginning of the experimental protocol, sham and castrated mice displayed similar body weight. Both groups gained weight during the protocol development. However, the weight gain was higher in mice fed the HFD as compared to the CD-fed group. Castrated and Sham mice on the HFD showed similar body weight gain. Similarly, testosterone treatment did not modify weight gain in HFD- or CD-fed mice. Castration effectively reduced endogenous androgen levels, as evidenced by the undetectable levels of testosterone in castrated animals. Castrated mice fed the CD and treated with testosterone propionate (5 mg/kg body weight) exhibited supraphysiological levels of testosterone, whereas castrated HFD-fed mice treated with the same dose of testosterone propionate (5 mg/kg body weight) exhibited testosterone levels similar to those observed in the sham group.

**TABLE 1:** Body weight and biochemical profile in the experimental groups of mice.

	CD Sham	CD Castrated	HFD Sham	HFD Castrated	CD Castrated + Testo	HFD Castrated + Testo
Body weight (g)	28.1 ± 0.4	26.7 ± 0.6	43.8 ± 0.4*	41.7 ± 0.8	28.9 ± 0.5	45.1 ± 0.3
Testosterone (ng/mL)	7.7 ± 0.7	0.9 ± 0.4*	6.4 ± 0.8	0.6 ± 0.2^#^	11.4 ± 1.1*	7.8 ± 1.2
Glucose (mg/dL)	79.8 ± 3.1	84.8 ± 2.8	184.3 ± 5.9*	141.4 ± 10.7^#^		
Insulin (ng/mL)	3.6 ± 0.2	3.4 ± 0.3	8.2 ± 0.6*	7.4 ± 0.5		
Total Cholesterol (mg/dL)	81.6 ± 3.5	80.1 ± 3.2	121.6 ± 6.6*	129.1 ± 6.5		
Triglyceride (mg/dL)	67.1 ± 5.4	45.3 ± 6.1*	106.3 ± 10.1*	57.3 ± 8.5^#^		

Values are expressed as mean ± SEM (n = 10–12). CD, control diet; HFD, high fat diet. Two-way ANOVA: *p < 0.05 vs. CD Sham; ^#^p < 0.05 vs. HFD Sham.

No differences in serum glucose were observed between sham and castrated mice fed the CD. However, HFD increased serum glucose in sham mice. Insulin and total cholesterol plasma levels were also increased in HFD-fed sham mice compared to CD-fed sham mice. Castration did not change increased serum glucose, insulin or total cholesterol levels induced by the HFD, but decreased triglyceride plasma levels in mice fed both the CD and HFD (Table 1). These results indicate that metabolic changes evoked by the HFD in young mice are not affected by either castration or supraphysiological levels of circulating testosterone.

### Castration Prevents Vascular Dysfunction in High Fat Diet-Fed Mice

**FIGURE 1:**
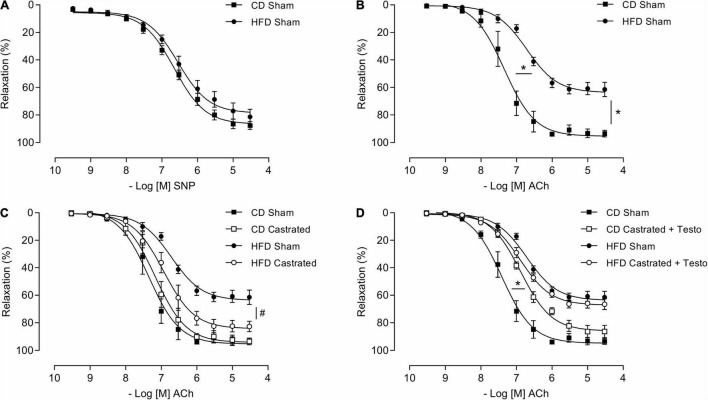
Testosterone contributes to vascular dysfunction in high fat diet (HFD)-fed mice. Concentration-response curves to sodium nitroprusside - SNP **(A)** and acetylcholine (Ach) were performed in aortic rings isolated from control diet (CD) sham and HFD sham mice **(B)**. ACh responses were also determined in aortic rings from castrated mice fed either a CD or HFD (CD castrated and HFD castrated, respectively) **(C)**, and CD castrated and HFD castrated mice treated with testosterone propionate (5 mg/Kg for 15 days) **(D)**. Data are expressed as mean ± SEM (*n* = 7 in all groups). *p < 0.05 *vs*. Sham; ^#^*p* < 0.05 *vs*. HFD Sham.

We then assessed the involvement of testosterone on vascular dysfunction in HFD-fed mice considering its independent association with cardiovascular complications. Castrated and sham-operated mice were fed the HFD and vascular function was evaluated. As shown in Figure 1A, no differences in SNP-induced vascular relaxation were observed between CD sham and HFD sham mice. In contrast, HFD reduced ACh-induced vascular relaxation in sham mice in comparison with CD-fed sham mice (Figure 1B). In mice fed the CD, there were no differences in ACh-induced vasodilatation between the sham and castrated groups. However, castration prevented HFD-induced vascular dysfunction (Figure 1C). Treatment of castrated mice maintained on the CD with testosterone propionate decreased ACh-induced vasodilation. The effects of castration on ACh vasodilation in HFD-fed mice, i.e., increased ACh vasodilatation, were lost upon testosterone propionate treatment (Figure 1D). Table 2 shows the maximum response and *p*D_2_ values.

**TABLE 2:** Emax and *p*D_2_ values of sodium nitroprusside and acetylcholine-induced relaxation in thoracic aorta arteries.

	CD Sham (*SNP*)	HFD Sham (*SNP*)	CD Sham (*ACh*)	HFD Sham (*ACh*)	CD Castrated (*ACh*)	HFD Castrated (*ACh*)	CD Castrated + Testo (*ACh*)	HFD Castrated + Testo (*ACh*)
Emax	86.6 ± 1.6	79.6 ± 2.4	95.3 ± 2.7	63.7 ± 1.7*	94.1 ± 2.5	84.2 ± 2.4^#^	85.8 ± 1.6	67.1 ± 1.3
*p*D_2_	6.65 ± 0.05	6.54 ± 0.08	7.34 ± 0.08	6.71 ± 0.07*	7.16 ± 0.07	6.93 ± 0.08	6.88 ± 0.05*	6.89 ± 0.05

Values are expressed as mean ± SEM (n = 7). CD, control diet; HFD, high fat diet; SNP, sodium nitroprusside; ACh, acetylcholine. Two-way ANOVA: *p < 0.05 vs. CD Sham; ^#^p < 0.05 vs. HFD Sham.

### Castration Prevents Vascular Oxidative Stress in High Fat Diet-Fed Mice

**FIGURE 2:**
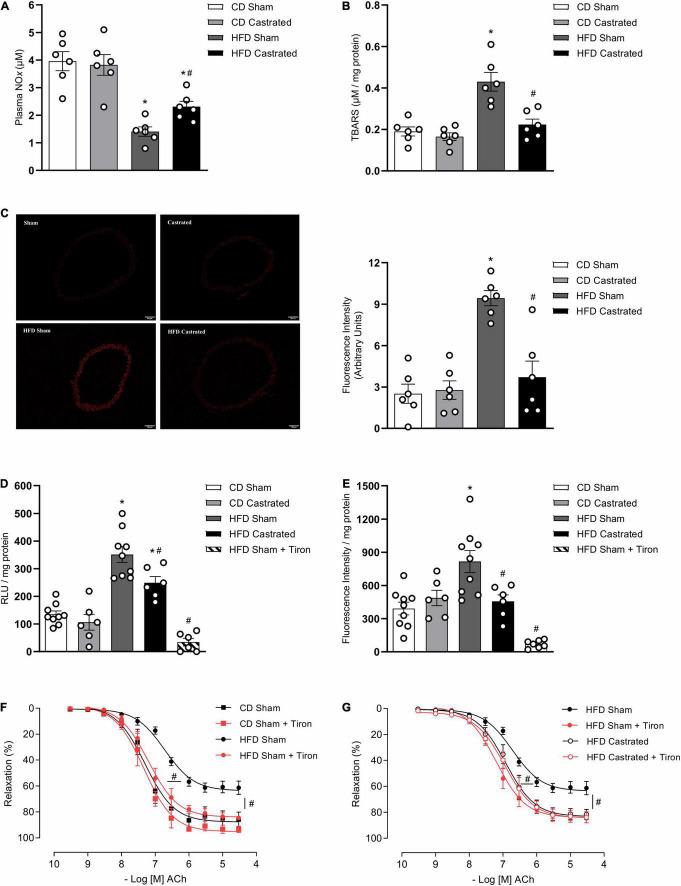
Testosterone contributes to vascular oxidative stress in high fat diet (HFD)-fed mice. Plasma NO*x* [**(A)** *n* = 6 for each experimental group], TBARS [**(B)** *n* = 6 for each experimental group], reactive oxygen species generation – measured by DHE [**(C)** *n* = 6 for each experimental group], superoxide anion generation – measured by lucigenin [**(D)** *n* = 6 or 9 for each experimental group], and hydrogen peroxide levels – determined by amplex red [**(E)** *n* = 6 or 9 for each experimental group] were evaluated in aortas isolated from mice of the different experimental groups. Concentration-response curves to acetylcholine (Ach) were performed in aortic rings isolated from control diet (CD) sham, HFD sham, and HFD castrated mice in the presence of Tiron (10^–4^ M) [**(F,G)** *n* = 7 in all groups]. Data are expressed as mean ± SEM. *p < 0.05 *vs*. CD Sham; ^#^*p* < 0.05 *vs*. HFD Sham.

We next determined whether testosterone contributes to increased vascular ROS generation in mice on the HFD. As expected and already reported, HFD decreased plasma NO*x* (Figure 2A) and increased aortic TBARS levels (Figure 2B). Basal levels of ROS, determined by DHE fluorescence intensity (Figure 2C), were significantly increased in arteries from HFD-fed sham mice. In addition, aortas from HFD sham mice exhibited increased generation of O_2_^–^ (Figure 2D) and H_2_O_2_ (Figure 2E), determined by lucigenin chemiluminescence and amplex red, respectively. Castration prevented increased vascular ROS induced by HFD. No differences were observed in ROS generation between sham and castrated mice on the CD. Similarly to castration, tiron improved ACh-induced vascular relaxation in HFD sham mice (Figure 2F). No additional effects were observed with the combination of tiron and castration, i.e., tiron did not further improve ACh vasodilation in HFD castrated mice (Figure 2G), indicating that both tiron and castration interfere with similar pathways. Table 3 shows the maximum response and *p*D_2_ values.

**TABLE 3:** Emax and *p*D_2_ values of acetylcholine-induced relaxation in thoracic aorta arteries incubated with vehicle or Tiron.

	CD Sham	CD Sham + Tiron	HFD Sham	HFD Sham + Tiron	HFD Castrated	HFD Castrated + Tiron
Emax	87.7 ± 3.1	95.1 ± 2.5	63.7 ± 1.7	83.9 ± 1.9^#^	83.2 ± 2.3^#^	84.2 ± 2.2
*p*D_2_	7.30 ± 0.10	7.33 ± 0.08	6.71 ± 0.07	7.19 ± 0.06^#^	6.92 ± 0.08^#^	6.95 ± 0.07

*Values are expressed as mean ± SEM (n = 7). CD, control diet; HFD, high fat diet. Two-way ANOVA: ^#^p < 0.05 vs. HFD Sham.*

### Castration Prevents Downregulation of the Antioxidant System in High Fat Diet-Fed Mice

**FIGURE 3:**
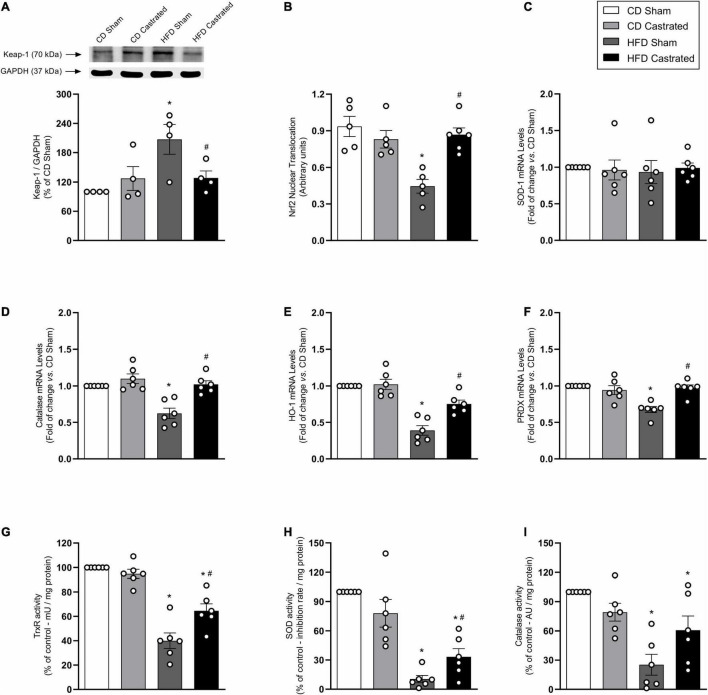
Testosterone contributes to downregulation of the nuclear factor E2–related factor 2 (Nrf2) antioxidant system in high fat diet (HFD)-fed mice. The experiments were performed in aortas isolated from the various experimental groups. Total levels of Keap-1 [**(A)** *n* = 4 for each experimental group]. Nuclear accumulation of Nrf2 [**(B)** *n* = 5 or 6 for each experimental group]. mRNA expression of genes regulated by Nrf2: SOD-1 [**(C)** *n* = 6 for each experimental group], catalase [**(D)** *n* = 6 for each experimental group], heme oxygenase-1 [**(E)** *n* = 6 for each experimental group] and peroxiredoxin-1 [**(F)** *n* = 6 for each experimental group]. Activity of antioxidant enzymes thioredoxin reductase [**(G)** *n* = 6 for each experimental group], SOD [**(H)** *n* = 6 for each experimental group] and catalase [**(I)** *n* = 6 for each experimental group]. Data are expressed as mean ± SEM. *p < 0.05 *vs*. CD Sham; ^#^*p* < 0.05 *vs*. HFD Sham.

Nuclear factor E2–related factor 2 signaling is a major regulator of cellular antioxidant systems. HFD increased vascular Keap-1 protein expression (Figure 3A). In addition, HFD decreased nuclear accumulation of Nrf2 (Figure 3B), which was associated with a decrease in mRNA expression of Nrf2-regulated enzymes, such as SOD-1 (Figure 3C), catalase (Figure 3D), heme oxygenase-1 (Figure 3E), peroxiredoxin (Figure 3F) as well as with decreased activity of the enzymes thioredoxin (Figure 3G), SOD (Figure 3H) and catalase (Figure 3I). These events were also prevented in castrated HFD-fed mice, indicating that Nrf2-regulated redox balance in the vasculature of obese mice is modulated by testosterone. No differences were observed in Nrf2 antioxidant system between sham and castrated mice on the CD.

### Castration Prevents Vascular Dysfunction in High Fat Diet-Fed Mice by Mechanisms That Involve Nuclear Factor E2–Related Factor 2 Activation

**FIGURE 4:**
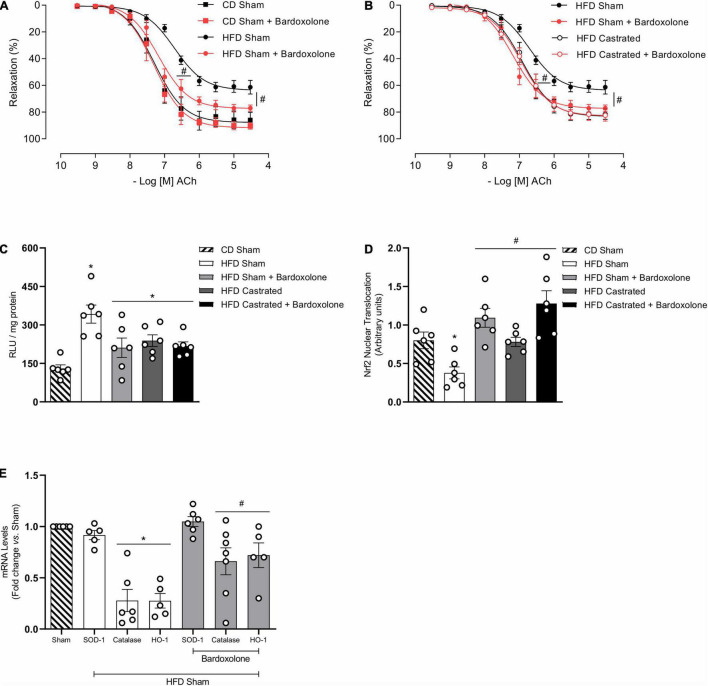
Castration prevents vascular dysfunction in high fat diet (HFD)-fed mice by mechanisms that involve nuclear factor E2–related factor 2 (Nrf2) system activation. Concentration-response curves to acetylcholine (ACh) were performed in aortic rings isolated from control diet (CD) sham, HFD sham and HFD castrated in the presence of Bardoxolone (10^–6^ M) [**(A,B)** *n* = 7 in all groups]. Superoxide anion generation – measured by lucigenin [**(C)** *n* = 6 for each experimental group], nuclear accumulation of Nrf2 [**(D)** *n* = 6 for each experimental group], and mRNA expression of genes regulated by Nrf2 [**(E)** *n* = 5 or 6 for each experimental group] were determined in aortas isolated from mice of the various experimental groups. Data are expressed as mean ± SEM. *p < 0.05 *vs*. CD Sham; ^#^*p* < 0.05 *vs*. HFD Sham.

Activation of the Nrf2 system by bardoxolone improved ACh-induced vascular relaxation in HFD-fed sham mice (Figure 4A). The effects of bardoxolone and castration on ACh vasodilation were similar in HFD-fed mice (Figure 4B), with no additional effects being observed in arteries from castrated mice incubated with bardoxolone. Bardoxolone did not change vascular reactivity in sham or castrated mice fed the CD. Bardoxolone increased nuclear accumulation of Nrf2 in vessels from HFD-fed sham mice (Figure 4C) and decreased HFD-induced vascular ROS generation (Figure 4D). Notably, no synergism was observed between the effects of bardoxolone and castration. In addition, bardoxolone improved catalase and heme oxygenase-1 mRNA expression (Figure 4E). Table 4 shows the maximum response and *p*D_2_ values.

**TABLE 4:** Emax and *p*D_2_ values of acetylcholine-induced relaxation in thoracic aorta arteries incubated with vehicle or Bardoxolone.

	CD Sham	CD Sham + Bardoxolone	HFD Sham	HFD Sham + Bardoxolone	HFD Castrated	HFD Castrated + Bardoxolone
Emax	87.7 ± 3.1	91.7 ± 2.2	63.7 ± 1.7	77.2 ± 1.8^#^	83.2 ± 2.3^#^	82.8 ± 2.3
*p*D_2_	7.30 ± 0.10	7.31 ± 0.06	6.71 ± 0.07	7.18 ± 0.07^#^	6.92 ± 0.08^#^	6.94 ± 0.06

*Values are expressed as mean ± SEM (n = 7). CD, control diet; HFD, high fat diet. Two-way ANOVA: ^#^p < 0.05 vs. HFD Sham.*

### Castration Prevents Increased NADPH Oxidase Expression in High Fat Diet-Fed Mice

**FIGURE 5:**
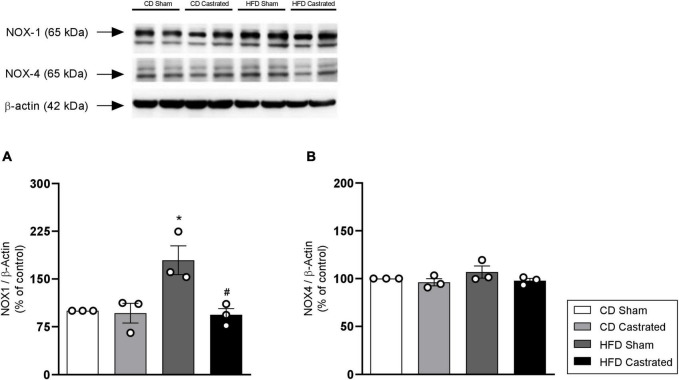
Testosterone contributes to increased vascular NOX1 expression in HFD-fed mice. The experiments were performed in aortas isolated from mice of the different experimental groups. Total levels of NOX1 **(A)** and NOX4 **(B)**, determined by western blot, were normalized by β-actin expression. Data are expressed as mean ± SEM (*n* = 3 in all groups). *p < 0.05 *vs*. CD Sham; ^#^*p* < 0.05 *vs*. HFD Sham.

NADPH oxidase is the main source of free radicals in the vasculature. HFD increased NOX1 expression in aortas, which was prevented in castrated HFD-fed mice (Figure 5A). No difference was observed in the antioxidant system between sham and castrated mice fed the CD. In addition, no difference was observed in NOX4 (Figure 5B) expression among the different experimental groups.

### NOX-1 Contribution to Testosterone-Induced Reactive Oxygen Species Generation and Endothelial Dysfunction

**FIGURE 6:**
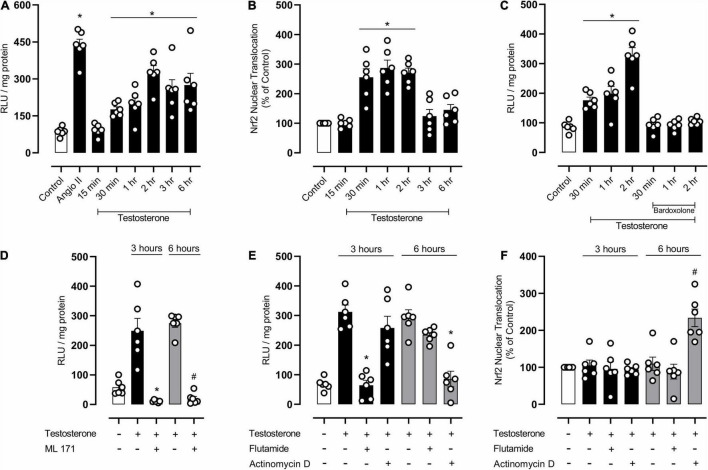
Testosterone induces a biphasic increase in reactive oxygen species (ROS) generation by mechanisms that involves NOX1 activation and downregulation of Nrf2 activity in endothelial cells. Superoxide anion generation was measured by lucigenin [**(A,C–E)** *n* = 6 or 7 for each experimental group] and nuclear accumulation of Nrf2 [**(B,F)** *n* = 6 for each experimental group]. Data are expressed as mean ± SEM. *p < 0.05 *vs*. Control; ^#^*p* < 0.05 *vs*. Testosterone.

Considering that testosterone modulates endothelial cell function as well as oxidative and inflammatory processes in endothelial cells, we further determined molecular mechanisms involved in testosterone-mediated endothelial dysfunction, by using EA.hy926 cells. Testosterone induced a time-dependent ROS generation in EA.hy926 cells (Figure 6A), with maximum generation occurring at 2 h. In addition, testosterone increased nuclear accumulation of Nrf2 from 30 min, but this increase was reduced after 3 h of stimulation with testosterone (Figure 6B). Incubation of EA.hy926 cells with bardoxolone prevented ROS generation (Figure 6C). Testosterone-induced ROS generation at 3 and 6 h was prevented by ML171, a NOX1 inhibitor (Figure 6D). To investigate the possible mechanisms (genomic and non-genomic) whereby testosterone induces ROS production at 3 and 6 h, ROS generation was determined in the presence of flutamide and actinomycin D. Pre-incubation with flutamide prevented testosterone-induced ROS generation at 3 h, but not at 6 h. On the other hand, incubation with actinomycin D prevented testosterone-induced ROS generation at 6 h, but not at 3 h (Figure 6E). Actinomycin D prevented the decrease in Nrf2 activity induced by testosterone. Testosterone-induced decrease in Nrf2 activity was not observed in the presence of flutamide (Figure 6F).

## Discussion

The present study shows that testosterone plays a critical role in the progressive vascular oxidative damage caused by obesogenic diets in young male mice, mediating a set of changes that are very similar to those previously described in adult males. Accordingly, we demonstrated that testosterone, via androgen receptors, induces NOX1-dependent oxidative stress and negatively impacts Nrf2/Keap1 signaling, one of the main protective responses to oxidative stress.

It is extensively known that testosterone does not contribute exclusively to the development of the male reproductive system and sexual maturation. Clinical and experimental evidence shows that testosterone also modulates cardiovascular homeostasis and produces important cardiac and vascular effects in physiopathological conditions, including obesity (Torres et al., 2007; Wang et al., 2011; Fui et al., 2014). Adult wistar rats fed a sucrose-rich diet exhibit increased vascular contractions to noradrenaline and this increase is prevented by castration (Vasudevan et al., 2006; Perez et al., 2009). In line with these observations, the present study shows that obesity in young male mice impairs vascular function, as evidenced by the decrease in acetylcholine-mediated vasodilation. Castration of these young animals also prevented obesity-associated endothelial dysfunction. The findings that testosterone replacement in castrated young obese mice impairs vasodilation, confirms that testosterone negatively influences vascular tone during obesity development in young mice.

Although the mechanisms by which obesity causes vascular dysfunction in adult are still not completely known, it is clear that this condition is associated with vascular oxidative stress, increased vasoconstriction and decreased vasodilation (Stapleton et al., 2008). Accordingly, our data clearly show that obesity induced by a high fat diet in young mice also increases free radical’s generation in the vasculature, increases lipid peroxidation and reduces NO bioavailability. In addition, castration prevents oxidative stress and improves endothelial function in arteries of obese mice, indicating that testosterone is pro-oxidant and contributes to vascular dysfunction in young obesity. This is in agreement with previous studies demonstrating that, in adult experimental models, testosterone increases ROS generation in VSMC via NADPH oxidase- (Chignalia et al., 2012) and mitochondrial respiratory chain-dependent mechanisms (Lopes et al., 2014).

Free radical’s levels are controlled by intracellular defense mechanisms that rely on the expression and activity of antioxidant enzymes. The Nrf2 system is essential for redox balance in the cardiovascular system. Nrf2 activation has vasoprotective effects via reduction of ROS and increased bioavailability of NO (Mann et al., 2007; Lopes et al., 2015). In adult obesity, the Nrf2 system is downregulated, since Nrf2 translocation to the nucleus is decreased in arteries of obese mice. In addition, expression of Keap-1, which negatively regulates Nrf2 degradation, maintaining Nrf2 in the cytosol, was increased in arteries from obese mice. Increased Keap-1 expression may account for the decreased vascular nuclear translocation of Nrf2. This is supported by studies showing decreased activity of Nrf2 in adipose tissue (Zamora-Mendoza et al., 2018) and renal cells of obese mice (Chin et al., 2013). In addition, mesenteric arteries of obese animals that do not express the leptin receptor exhibit decreased Nrf2 activity, which contributes to oxidative stress and increased vasoconstriction. Treatment of these animals with the Nrf2 activator L-sulforaphane reduces ROS and improves vascular function (Alves-Lopes et al., 2016). These data further support our results showing that decreased Nrf2 activity impairs vascular function and that activation of the Nrf2 system by bardoxolone prevents high fat diet-induced vascular dysfunction in young obese mice.

Castration increases Nrf2 activity and the expression and activity of antioxidant enzymes in arteries of obese mice, clearly indicating that testosterone negatively regulates Nrf2 signaling during transition from early to middle adulthood, when most body mass gain occurs. Considering that fat deposition early in life accounts for increased cardiovascular risk in adulthood, the effects of testosterone, demonstrated in our study, may represent an important mechanism mediating this process.

A link between Nrf2 and testosterone has been previously reported in adult experimental models. Nrf2 knockout mice exhibit increased ROS generation and fluctuations in plasma testosterone levels due to Leydig cell deficiency (Chen et al., 2015). Recent data from our group showed that hyperglycemia, a condition found in diabetes mellitus, reduces Nrf2 activity, increases the activity of pro-oxidant enzymes and ROS generation, leading to vascular (pudendal arteries) dysfunction. Considering that this vascular bed is highly responsive to the actions of testosterone (Alves-Lopes et al., 2016), this reinforces the hypothesis that blood vessels are modulated by redox mechanisms sensitive to testosterone even during fat accumulation early in life.

Arteries of young obese mice exhibited increased expression of NOX1, a NAD(P)H oxidase isoform, and, interestingly, castration prevented this increase. Accordingly, it has been reported that testosterone increases the expression of NADPH oxidase subunits in VSMC, increasing ROS generation and cell migration (Chignalia et al., 2012). We sought to further explore the mechanisms activated by testosterone that culminate in endothelial dysfunction and subsequent vascular dysfunction. In cultured endothelial cells, testosterone increased ROS generation in a time-dependent manner; initially by non-genomic actions and activation of androgen receptors and later by genomic effects. Testosterone also decreased Nrf2 activity by mechanisms that rely on gene transcription. Similar to the present findings, it was reported that ROS generation induced by testosterone depends on early-activated genomic mechanisms and also on lately-activated non-genomic mechanisms (Chignalia et al., 2015).

In conclusion, the present study demonstrated that testosterone downregulates the Nrf2 antioxidant system, favoring NOX1 activity, ROS accumulation and vascular dysfunction in HFD-fed young obese mice. These findings indicate that testosterone constitutes an important mediator of the progressive vascular oxidative damage caused by obesogenic diets in young male mice, favoring a vascular oxidative status that is similar to that observed in adult obesity.

## Data Availability Statement

The raw data supporting the conclusions of this article will be made available by the authors, without undue reservation.

## Ethics Statement

The animal study was reviewed and approved by Ethics Committee on Animal Use (CEUA) of the University of São Paulo, Ribeirão Preto, Brazil (Protocol no 206/2016).

## Author Contributions

RC, NL, FC, and RT participated in the design of the study, provided the reagents and analytical tools, and wrote the manuscript. RC, RA-L, JA, CS, and FM performed the experiments and data analysis. All authors contributed to the article and approved the submitted version.

## Conflict of Interest

The authors declare that the research was conducted in the absence of any commercial or financial relationships that could be construed as a potential conflict of interest.

## Publisher’s Note

All claims expressed in this article are solely those of the authors and do not necessarily represent those of their affiliated organizations, or those of the publisher, the editors and the reviewers. Any product that may be evaluated in this article, or claim that may be made by its manufacturer, is not guaranteed or endorsed by the publisher.
