# Supplementary figures and images for: Testosterone Contributes to Vascular Dysfunction in Young Mice Fed a High Fat Diet by Promoting Nuclear Factor E2–Related Factor 2 Downregulation and Oxidative Stress
Source: Front Physiol. 2022 Mar 8;13:837603. doi: 10.3389/fphys.2022.837603 (PMC8958040; doi:10.3389/fphys.2022.837603)

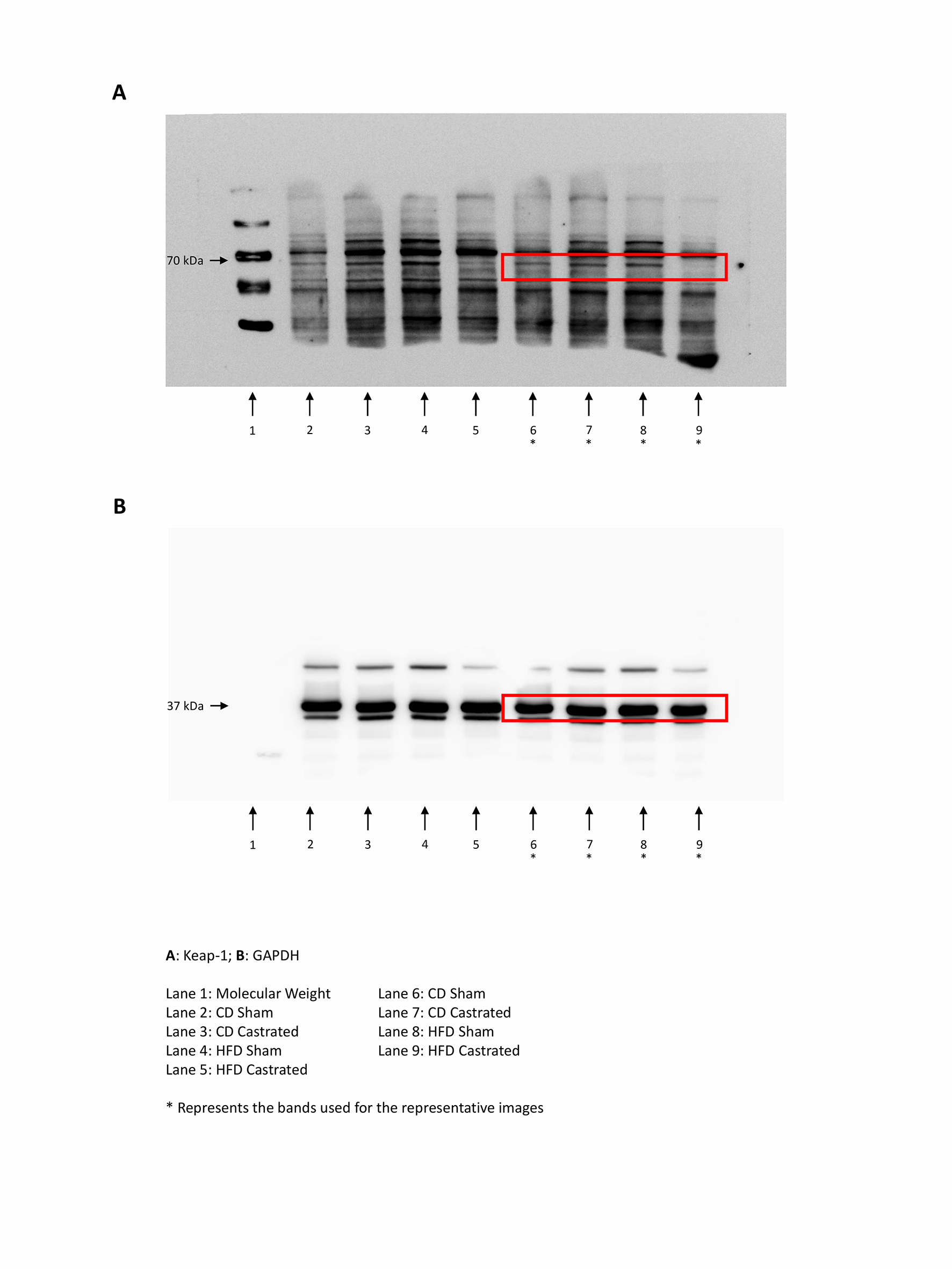

Supplement: Supplementary file 1 [file Image_1.tif]

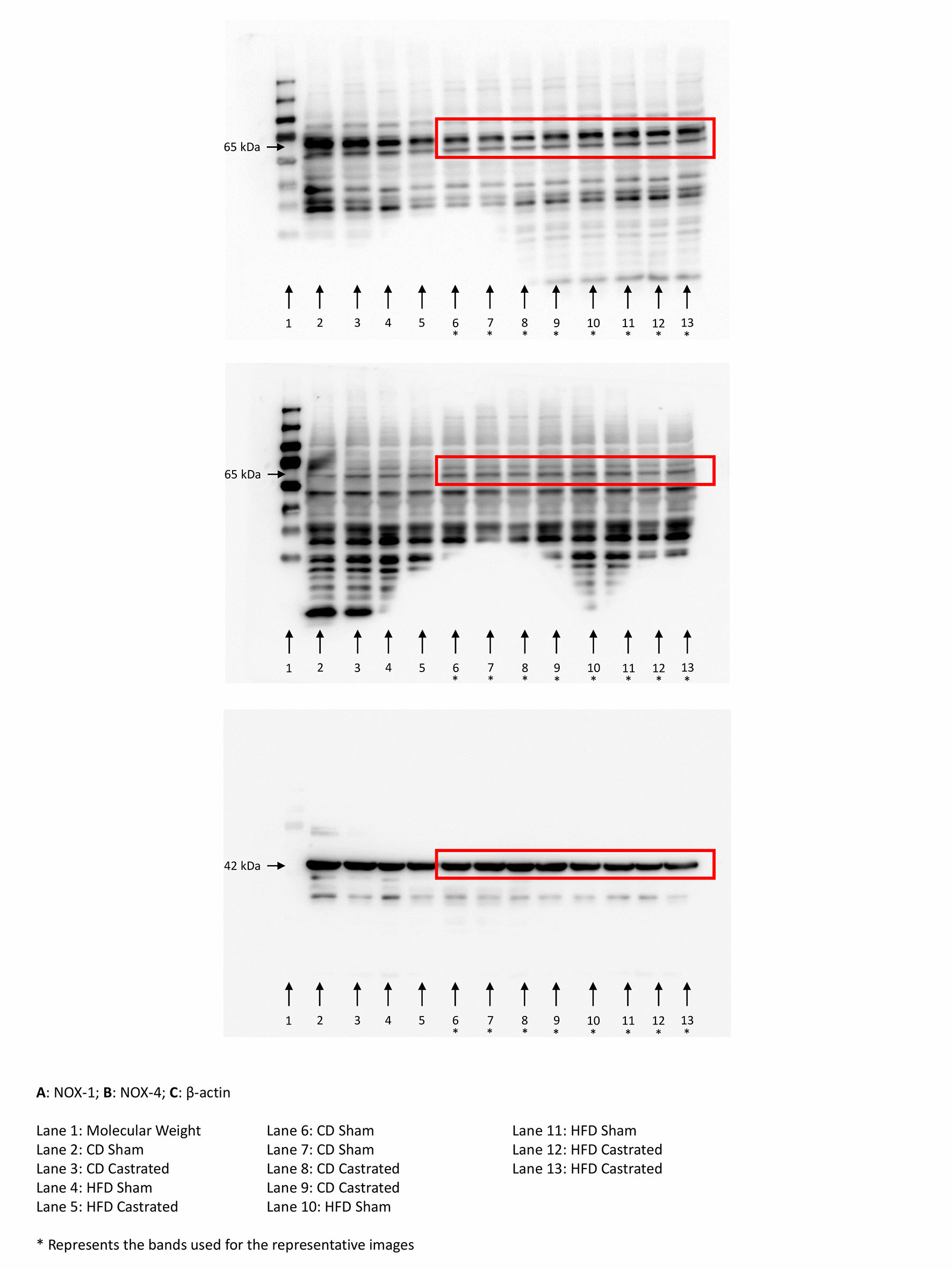

Supplement: Supplementary file 2 [file Image_2.tif]
